# COVID-19 prophylaxis with immunoglobulin Y (IgY) for the world population: The critical role that governments and non-governmental organizations can play

**DOI:** 10.7189/jogh.12.03080

**Published:** 2022-12-03

**Authors:** Lyn R Frumkin, Michaela Lucas, Michael Wallach, Curtis L Scribner, Tom St John, Daria Mochly-Rosen

**Affiliations:** 1 SPARK at Stanford, Stanford University, School of Medicine, Stanford, California, USA; 2 Medical School, The University of Western Australia, Perth, Western Australia, Australia; 3 University of Technology Sydney, Sydney, New South Wales, Australia; 4 SPARK Sydney, Sydney, New South Wales, Australia; 5 Independent Regulatory Consultant, Oakland, California, USA; 6 Department of Chemical and Systems Biology, Stanford University, School of Medicine, Stanford, California, USA; 7 SPARK Global, Stanford University, School of Medicine, Stanford, California, USA

Fewer than 25% of people in low-income countries are estimated to have received at least one COVID-19 vaccine dose to date, in contrast to 68% of the world population [1]. Besides the local culture and social conditions that contribute to vaccine hesitancy, multiple inequities inherent to global public health complicate the ability to vaccinate against COVID-19 in lower- and middle-income countries; these include the cost of antiviral drug development, difficulties in vaccine manufacturing and distribution, non-local production, vaccine nationalism, and failure of virus- and vaccine-induced immunity to prevent transmission. There are pressing practical and ethical reasons for achieving vaccine equity [2]. However, as this goal remains elusive, there is also a critical need to develop safe, effective, easy-to-produce, and inexpensive treatments that can complement vaccinations and that can be produced locally to reduce the risk of acquiring SARS-CoV-2 infection globally, particularly in low- and middle-income countries. Viral variation, even in areas with high vaccine uptake, further complicates this challenge.

The nasal mucosa is the primary entry route for SARS-CoV-2, given that it contains a high level of the human angiotensin-converting enzyme 2 (hACE2) receptor used by the virus to gain cellular entry [3]. Antibodies against the SARS-CoV-2 receptor-binding domain can compete with viral binding to the hACE2 receptor, making the nasal mucosa an excellent site as a critical barrier to reducing SARS-CoV-2 entry. Studies (mainly in animal models) have examined methods for anti-COVID-19 intranasal prophylaxis that include polymer barriers, active vaccines, existing antiviral drugs, inhibitors of protease-induced activation of the virus, antiseptics, antimicrobial agents, and antibodies [4-11]. An optimal agent for intranasal prophylaxis would incorporate several key properties, including a broad, robust, and variant-insensitive specificity, a simple and low-cost manufacturing process able to be used in low-resource settings, and stability with a long product life.

## PASSIVE IMMUNIZATION AND IMMUNOGLOBIN Y

Passive immunization with parenterally-given immunoglobulin G has a long history of effectiveness in preventing human infectious diseases caused by viruses [12]. Intranasal antibody prophylaxis has also been an especially effective means to protect against multiple viral pathogens [13]. Egg yolk antibodies called immunoglobulin Y (IgY) have been effective in preventing disease transmission when given prophylactically in both animal models and human clinical settings of viral and bacterial diseases (as reviewed in [14]).

IgY antibodies, which do not activate the human complement system or bind the Fc receptor on immune cells, are known for their favourable safety profile. Overall, available data suggest that IgY antibodies given by non-parenteral administration do not have unwanted off-target pro-inflammatory effects and are non-toxic to humans, allowing for potential clinical applications in diverse populations and diseases [14,15], including the elderly, the immunocompromised, and children. IgY prophylaxis may also be valuable when used with personal protective equipment for individuals at increased risk of infection.

**Figure 1:**
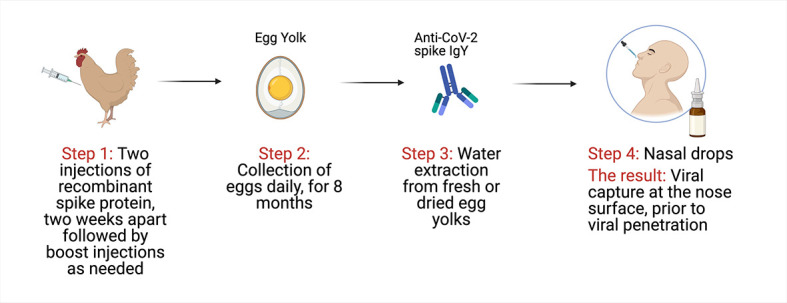
Anti-SARS-CoV-2 IgY as passive immunization against COVID-19 (modified from [23] under CC BY 4.0 license).

IgY is cheap, simple, and fast to produce [16]. The high yield of IgY per egg, rapid scale-up, and mass production at low cost (including in low-resource settings) make this a very practical approach as a potential passive immunization against COVID-19. After a laying hen is immunized with recombinant antigen, eggs can be produced for 8-10 months at a rate of nearly one egg a day, each containing up to 100 mg of IgY. This yield can be up to five times higher when using specific-pathogen-free hens. IgY purification can be achieved by a simple water extraction process (**Figure 1**). We reported a step-by-step protocol for IgY purification in low- and middle-income countries using inexpensive, readily available materials in place of costly, specialized laboratory equipment and chemicals [17].

## CONCLUSION

The global death toll from COVID-19 is now over six million, with estimates of excess deaths associated with COVID-19 at least double that figure during 2020 and 2021 alone [18]. The impact of COVID-19, including long COVID, is sobering, regardless of a country’s economic status. In the United States, COVID-19 was the third leading cause of death (after heart disease and cancer) in 2021 and has caused the biggest drop in life expectancy since World War II [19]. Similar patterns have emerged in Europe [19]. Urgent calls for global COVID-19 “vaccine-plus” approaches have been made [20]. Yet, global inequities that demand novel and local approaches to treatments are most critical for low- and middle-income countries. For example, the Africa Centres for Disease Control and Prevention (CDC) cautioned that less than 1% of vaccines on the continent are manufactured locally, which precludes an efficient response to pandemics such as COVID-19 [21].

IgY from hens immunized with inactivated SARS-CoV-2, recombinant S protein, or N protein can neutralize the virus in vitro. Additionally, intranasally administered IgY antibodies directed to the receptor-binding domain of SARS-CoV-2 protected hamsters [10] and mice [22] challenged with the virus. We have recently reported that anti-SARS-CoV-2 neutralizing hen IgY, which is effective against several variants of concern in vitro that indicate a diverse and polyclonal response, had an excellent safety profile in humans without systemic absorption when used as intranasal drops in a phase 1 clinical trial [23]. The large-scale, local, ecologically sound, and animal-friendly technology of production and affordability of high-titer anti-SARS-CoV-2 IgY make it attractive for further studies to provide global protection in resource-limited environments. Furthermore, because current variants of concern have significantly reduced vaccine effectiveness, and future variants may cause potentially more serious and lethal diseases, clinical trials can now define whether IgY may be a rapid means to halt the pandemic more broadly than is presently possible.

**Figure:**
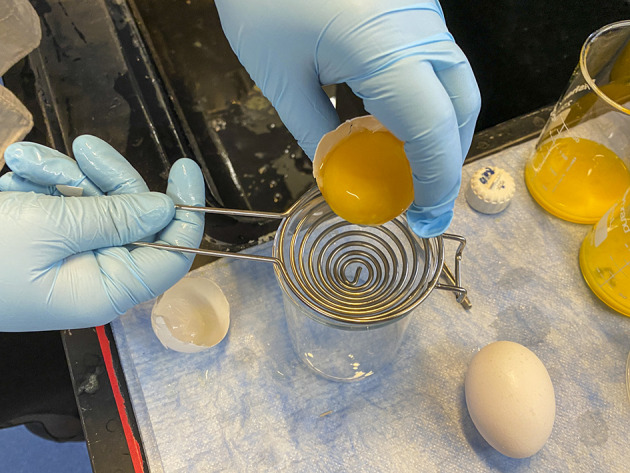
Photo: Extraction and purification of immunoglobulin Y (IgY). Source: Ana Koperniku and Allison Jia, Stanford University. Used with permission.

Public and private funding of COVID-19 drug and vaccine development has been significant. Moreover, a wide range of actions to enable more equitable global access to COVID-19 therapeutics has been proposed. Yet, a difficult route to economic benefit has likely hampered the commercial development of IgY therapeutics by industry. This is a call to action for private, governmental, for-profit, and non-profit sectors to take on the challenge.
